# Self-reported physical activity correlates in Swedish adults with multiple sclerosis: a cross-sectional study

**DOI:** 10.1186/s12883-017-0981-4

**Published:** 2017-12-01

**Authors:** Elisabeth Anens, Lena Zetterberg, Charlotte Urell, Margareta Emtner, Karin Hellström

**Affiliations:** Department of Neuroscience, Section for Physiotherapy, Box 593 Uppsala University, 751 24 Uppsala, Sweden

**Keywords:** Exercise, Multiple sclerosis, Physical therapy, Rehabilitation, Self-efficacy

## Abstract

**Background:**

The benefits of physical activity in persons with Multiple Sclerosis (MS) are considerable. Knowledge about factors that correlate to physical activity is helpful in order to develop successful strategies to increase physical activity in persons with MS. Previous studies have focused on correlates to physical activity in MS, however falls self-efficacy, social support and enjoyment of physical activity are not much studied, as well as if the correlates differ with regard to disease severity. The aim of the study was to examine associations between physical activity and age, gender, employment, having children living at home, education, disease type, disease severity, fatigue, self-efficacy for physical activity, falls self-efficacy, social support and enjoyment of physical activity in a sample of persons with MS and in subgroups with regard to disease severity.

**Methods:**

This is a cross-sectional survey study including Swedish community living adults with MS, 287 persons, response rate 58.2%. The survey included standardized self-reported scales measuring physical activity, disease severity, fatigue, self-efficacy for physical activity, falls self-efficacy, and social support. Physical activity was measured by the Physical Activity Disability Survey – Revised.

**Results:**

Multiple regression analyzes showed that 59% (F(6,3) = 64.9, *p* = 0.000) of the variation in physical activity was explained by having less severe disease (β = −0.30), being employed (β = 0.26), having high falls self-efficacy (β = 0.20), having high self-efficacy for physical activity (β = 0.17), and enjoying physical activity (β = 0.11). In persons with moderate/severe MS, self-efficacy for physical activity explained physical activity.

**Conclusions:**

Consistent with previous research in persons with MS in other countries this study shows that disease severity, employment and self-efficacy for physical activity are important for physical activity. Additional important factors were falls self-efficacy and enjoyment. More research is needed to confirm this and the subgroup differences.

## Background

In persons with MS (PwMS) the positive effects of physical activity (PA) are considerable. Reviews have shown positive effects on e.g. muscle strength, aerobic capacity, fatigue, quality of life and depression [1–5]. However, the benefit of exercise for populations with severe disability requires further investigation [3]. Despite the evidence of multiple health benefits in PwMS the level of PA is low [6].

Knowledge about factors that correlate to PA is helpful in order to develop successful strategies to increase PA in PwMS. Previous studies show contradictory results regarding the influence of background factors on level of PA, such as age [7, 8], gender [8, 9], and having children living at home [7, 10]. A recent systematic review showed that employment status and educational level were consistent correlates of PA [11]. This review also showed that persons with greater disability are less physically active compared to those with a milder disease, but contradictory results were found regarding the influence of fatigue on PA [11]. Self-efficacy for PA, defined as the conviction that one can successfully execute the behavior required to produce a desired outcome [12] has shown to consistently facilitate PA [11]. Falls self-efficacy defined as the degree of efficacy (i.e. self-confidence) to avoid a fall [13], enjoyment of PA, and social support for PA are not extensively studied in PwMS.

A severe disease leads consistently to difficulties being physically active [6, 8, 9]. However, populations with more severe disability requires further investigation since MS participants in exercise interventions generally have a mild to moderate level of disability [3]. To get a better understanding of factors explaining PA in different subgroups and more useful results, it is of importance to investigate factors correlating to PA in PwMS with different disease severities.

The aim of this study was to examine the multivariate association between PA and age, gender, employment, having children living at home, education, type of MS, disease severity, fatigue, self-efficacy for PA, falls self-efficacy, social support and enjoyment of PA in PwMS. Subgroup analyses with regard to disease severity were also performed.

## Methods

### Design

This is a cross-sectional survey study.

### Participants

Adults with a diagnosis of MS were recruited from the Swedish Multiple Sclerosis Registry (McDonald and/or Poser criteria). The register stratifies four types of MS; relapsing remitting, secondary progressive, primary progressive and progressive relapsing. Inclusion criteria were: having MS, age between 18 and 80 years, participants living in the county of Uppsala. All registered participants living in the county of Uppsala were invited to participate (502 subjects). Exclusion criteria were: not understanding Swedish (*n* = 1), not living independently (n = 1), having another neurological disease (n = 1), not being able to answer the survey (n = 1). Five participants were also excluded due to not having the required diagnosis. The final study cohort consisted of 287 subjects (response rate 58.2%). In total there were 84 men (29.3%) and 203 women (70.7%), giving a female-to-male ratio of 2.42:1. Mean age was 51.5 (SD 13.5) years. The female-to-male ratio for the 206 subjects who did not respond to the invitation was 2.38:1. Non-respondents were slightly younger, mean age 49.0 (SD 13.4) years, *p* = 0.048.

### Procedure

A self-assessment questionnaire, an informatory letter, a consent form, and a stamped reply envelope were sent out by surface mail. The questionnaire was divided in two parts, with the second part sent out 2 weeks after a reply with answers to the first part of the questionnaire was received. Two reminders were sent to participants who did not answer either of the parts within 3 weeks. All participants provided written informed consent to participate. The study was approved by the Regional Ethical Review Board, Uppsala, Sweden, D-no, 2010/278.

### Outcome measures

For all measurements Swedish language versions and psychometrically sound measures were used. The amount of *PA* during the previous week was measured using the Physical Activity Disability Survey – Revised (PADS-R) [14]. The survey included six subscales; exercise, leisure time PA, general activity, therapy, employment and wheelchair use. The amount of PA during the previous week was reported for each scale. The subscale ratings were summed to give a total PADS-R. A higher score indicates more PA. *Disease severity* was measured using the Multiple Sclerosis Impact Scale (MSIS-29) [15]. The physical scale was used to classify the participants into two groups, indicating level of disease impact defined as ≤50 (minimal and mild disease), and >50 (moderate and severe disease) [16]. *Activity limitation* was measured using the ACTIVLIM questionnaire [17]. *Fatigue* was measured with the Fatigue Severity Scale (FSS) [18]. *Self-efficacy for PA* was measured using the Exercise Self-Efficacy Scale (ESES) [19]. *Falls self-efficacy* was measured using the Falls Efficacy Scale Swedish version (FES(S)) [20]. *Social support from family for PA* was measured using Social Influences on Physical Activity (SIPA) subscale influence from family [21]. The MSIS-29, ACTIVLIM, FSS, ESES, FES(S), and SIPA are described in detail in a previous descriptive study [9]. *Enjoyment of PA* was measured using three statements (developed by our research group) about experience during, or shortly following physical activity of at least 10 min duration (e.g. walking). “I experience that it is fun to be physically active”, “I experience a feeling of wellbeing when I am physically active”, “I feel happy with myself when I am physically active”. The answers were graded on a visual scale (from 0 to 5), and were added to a total score ranging from 0 to 15, with 15 being the highest level of enjoyment. Questions about background variables were also included in the questionnaire.

### Data analysis

Data was analyzed using IBM SPSS Statistics 23. The minimum sample size was estimated as 50 + 8 k, where k is the number of predictors, which results in a minimum sample size of 154 [22]. Missing data was handled as previously described [9]. Not all subjects answered all questions, hence the totals in the tables may differ. Forced entry multiple regression analysis was used to investigate factors that might influence PA. To evaluate the influence on PA with only the most important variables an additional regression analysis was performed, including all independent variables, with a standardized beta p- value of <0.2. No multicollinearity in the regression analysis was found by screening a correlation matrix of all included variables (*r* < 0.8) and by evaluating the variance inflation factors and tolerance statistics, except for in one case (See Discussion). There was no autocorrelation found with the Durbin-Watson test. The assumption of linearity, homoscedasticity and normally distributed residuals were met when checking the histogram and normal probability plots of the residuals. An acceptable level of outliers was found by evaluating the percentage of standardized residuals with an absolute value of greater than 2 (<5%) or 2.58 (<1%). The level of significance was set at *p* < 0.05.

## Results

Background variables for the 287 participants are described in Table 1. MS subtype was relapsing remitting in 135 (47.0%) and progressive in 146 (50.9%) persons. The mean PA level was 0.18 (SD 1.47). Thirty persons (10.5%) could not walk. For 148 (51.6%) persons walking distance outdoors was more than 500 m.

**Table 1:** Sample cohort description

Description of the cohort, n (%) or mean ± SD or median (quartiles)			
	ALL (*n* = 287)	Minimal/Mild MS (*n* = 204)	Moderate/Severe MS (*n* = 77)
Age (years)	51.5 ± 13.5	49.4 ± 13.6	57.5 ± 11.2
Gender			
Men	84 (29%)	46 (23%)	35 (46%)
Women	203 (71%)	158 (78%)	42 (55%)
Employment			
Yes	130 (45%)	116 (57%)	10 (13%)
Children at home			
Yes	71 (25%)	61 (30%)	7 (9%)
Tertiary education			
Yes	124 (43%)	96 (47%)	26 (34%)
Type of MS			
Relapsing remitting	135 (47%)	120 (59%)	12 (16%)
Secondary progressive	104 (36%)	59 (29)	43 (56%)
Primary progressive	32 (11%)	16 (7.8%)	16 (21%)
Progressive relapsing	10 (3.5%)	4 (2.0%)	5 (6.5%)
Subtype not known	6 (2.1%)	5 (2.5%)	1 (1.3%)
Progressive MS			
Yes	146 (51%)	79 (39%)	64 (83%)
Duration (years)^a^	11 (6-18)	9.5 (5-17)	13 (8-23)
MSIS-29			
Physical	28 (8-53)	18 (4-32)	66 (58-81)
Psychological	28 (11-47)	22 (8-36)	44 (31-64)
Physical activity ^b^	0.18 ± 1.47	0.75 ± 1.25	- 1.23 ± 0.99

*MSIS-29* Multiple Sclerosis Impact Scale. Minimal & Mild MS = MSIS-29 physical scale ≤50. Moderate & Severe MS = MSIS-29 physical scale >50

^a^Duration = time since diagnosis. ^b^ Physical activity was measured using the Physical Activity Disability Survey – Revised

### Factors explaining physical activity for all participants

Including all participants and all independent factors a model including low physical disease severity, being employed, high falls self-efficacy, and high self-efficacy for PA explained 57.6% of the variation in PA. When recalculating to include only the most important factors, also enjoyment of PA remained significant, resulting in 59% of the variation in PA being explained (Table 2).

**Table 2:** Physical activity regression models for all persons

All persons, multiple regression, physical activity (PA) dependent variable^a^				
	All factors (*n* = 250)		Factors with *p* < 0.20 (*n* = 267)	
Independent variables	β	*P*-value	β	*P*-value
Age	- 0.05	0.320	n.a.	n.a.
Gender	0.00	0.987	n.a.	n.a.
Employment	0.25	0.000*	0.26	0.000*
Children at home	- 0.03	0.473	n.a.	n.a.
Education	0.01	0.870	n.a.	n.a.
Progressive MS	- 0.03	0.541	n.a.	n.a.
MSIS-29 physical	- 0.30	0.002*	- 0.30	0.001*
MSIS-29 psychological	0.00	0.948	n.a.	n.a.
Fatigue	0.09	0.112	0.07	0.148
Self-efficacy for PA	0.13	0.035*	0.17	0.002*
Falls self-efficacy	0.21	0.011*	0.20	0.012*
Social support, family	0.02	0.585	n.a.	n.a.
Enjoyment of PA	0.08	0.120	0.11	0.021*
	R^2^ = 0.58, F(13,2) = 27.0, *p* = 0.000		R^2^ = 0.59, F(6,3) = 64.9, *p* = 0.000	

β = Standardized beta coefficient. * = *p* < 0.05. *R* ^*2*^ adjusted R square, *MSIS-29* Multiple Sclerosis Impact Scale

^a^Physical activity was measured using the Physical Activity Disability Survey – Revised

### Factors explaining physical activity for persons with different disease severity

In participants with minimal and mild MS (MSIS-29 physical scale ≤50) being employed, low activity limitations, high self-efficacy for PA and high enjoyment of PA explained 39.1% of the variation in PA (Table 3). In participants with moderate and severe MS (MSIS-29 physical scale <50), 41.3% of the variation in PA was explained by high self-efficacy for PA (Table 3).

**Table 3:** Physical activity regression models for persons with different disease severity

Multiple regression, physical activity (PA) dependent variable^a^				
	Minimal & Mild MS		Moderate & Severe MS	
	Factors with *p* < 0.20 (*n* = 196)		Factors with *p* < 0.20 (*n* = 73)	
Independent variables	β	*P*-value	β	*P*-value
Age	n.a.	n.a.	n.a.	n.a.
Gender	0.05	0.425	- 0.08	0.381
Employment	0.34	0.000*	0.15	0.132
Children at home	- 0.05	0.434	n.a.	n.a.
Education	n.a.	n.a.	n.a.	n.a.
Progressive MS	n.a.	n.a.	n.a.	n.a.
Activity limitation	0.26	0.000*	0.26	0.059
MSIS-29 psychological	n.a.	n.a.	n.a.	n.a.
Fatigue	n.a.	n.a.	0.16	0.099
Self-efficacy for PA	0.16	0.016*	0.25	0.027*
Falls self-efficacy	n.a.	n.a.	0.24	0.110
Social support, family	n.a.	n.a.	- 0.06	0.552
Enjoyment of PA	0.13	0.043*	n.a.	n.a.
	R^2^ = 0.39, F(5,2) = 26.1, *p* = 0.000		R^2^ = 0.41, F(5,7) = 11.1, *p* = 0.000	

β = Standardized beta coefficient. * = *p* < 0.05. *R* ^*2*^ adjusted R square, *MSIS-29* Multiple Sclerosis Impact Scale

Minimal & Mild MS = MSIS-29 physical scale ≤50. Moderate & Severe MS = MSIS-29 physical scale >50

^a^Physical activity was measured using the Physical Activity Disability Survey – Revised

## Discussion

Self-efficacy for PA consistently explained PA in the entire cohort and in all subgroups. This is consistent with previous results [11, 23]. This study confirms the importance of self-efficacy for PA also in persons with moderate and severe MS, categorized from MSIS-29. Unfortunately little research has focused on persons with moderate and severe MS. However, in a sample of 43 persons with moderate to severe MS, with an Expanded Disability Status Scale of 6.0 to 8.0, high general self-efficacy was associated with high levels of PA [24].

Falls self-efficacy, the degree of self-efficacy to avoid a fall, correlated to PA in the entire cohort. Our result is supported by a few previous studies. An exercise intervention led to increased confidence in performing activities without falling in PwMS [25]. High concerns about falling were associated with activity curtailment in PwMS [26]. The results of a review on interventions in PwMS to improve balance, indicates that programs incorporating gait, balance and functional training, especially when focusing on a high volume of challenging balance exercises, may lead to the greatest benefit in balance (and therefore potentially falls) outcomes [27].

Enjoyment of PA was associated with PA in the entire cohort, and in persons with minimal and mild MS. Experiencing enjoyment following exercise increased adherence to an exercise intervention in PwMS [28]. Most studies on correlates to PA in PwMS did not include enjoyment. However, a review showed that intrinsic motivation, or being active for the pleasure it brings, was the type of motivation that most strongly predicted long-term exercise adherence [29]. Little is known about factors that could increase enjoyment. Teixeira et al. [29] suggest e.g. emphasizing fun and skill improvement. Another review showed that experienced pleasure depends on PA intensity [30]. In addition, factors such as physical and social environment during PA might be important. The affective response during, and after PA, is probably important to consider when encouraging PA in PwMS.

In this cohort social support from family did not explain PA. Social support was inconsistently associated with PA in a previous review [11]. Possibly a more suitable questionnaire with focus on social support for PA in persons with disabilities could be of value.

Our results showed that low physical disease severity measured with MSIS-29 correlated to PA in the entire cohort. In line with this, a consistent correlate of PA in PwMS in a recent systematic review was disability level [11]. When analyzing subgroups from MSIS-29 the physical scale, ACTIVLIM was used as a measure of disease severity, which was highly correlated to MSIS-29 in this cohort. Having low activity limitations also explained a high level of PA in persons with minimal and mild MS, and probably also in moderate to severe MS, but was not significant in this small subgroup (Table 3). Other disease related factors measured in this study were having progressive MS, level of fatigue and MSIS-29 psychological scale. None of these were found to explain PA level in this cohort. In accordance with our result, Sterber et al. [11] reported in a review that type of MS, and fatigue, were not consistent correlates to PA. Moreover, changes in PA were not associated with fatigue in the 2,5 year longitudinal study by Motl et al. [23].

We found that the second most important factor in the entire cohort was being employed, with a standardized beta of 0.26. Being employed also explained level of PA in persons with minimal and mild MS. This is in line with findings in previous studies that higher levels of PA were consistently observed in PwMS who were employed [11]. One explanation is that persons who are employed are physically active at work, and when travelling to and from work.

Educational level did not influence the level of PA in this cohort which is in contrast to a previous review in PwMS [11]. However, this was based on results from studies in the USA, and one explanation could be cultural differences. No previous study on correlates to PA in PwMS in Sweden was found. It has been shown in a large Swedish study of the general population that education ≥12 years is associated with lower PA [31]. Persons with high education often have sedentary occupations. Measures of PA may include PA at work in varying amounts, which can also explain the differing results.

We studied gender differences in this cohort and found that men were less physically active than women and more physically affected by the disease [9]. However, in the regression analysis no association was found between gender and PA. This might be explained by men having more severe disease. Similar results were found in another sample of PwMS, where a gender difference in daily step counts was found, but when considering level of disability the difference disappeared [32].

The result for persons with minimal and mild MS, is more representative to the MS population due to the larger sample and the more similar results, all independent variables except falls self-efficacy were explaining PA in both the whole sample and in persons with minimal and mild MS (Tables 2 and 3). Falls self-efficacy was not important, probably due to less limited balance in persons with minimal and mild MS. Only one factor, self-efficacy for PA explained PA in persons with moderate and severe MS, probably more factors would become significant in larger samples. Maybe other not measured factors such as accessibility to exercise facilities, and support from others to perform activities would influence the possibility to be physically active in persons with moderate and severe MS.

In this study a non-respondent bias was found, since the non-respondents were slightly younger. Despite this finding the mean age of 51.5 years in this cohort is similar to the mean age of 52.6 years in a nationwide Swedish MS study (*n* = 17,485) [33]. Our female-to-male ratio was 2.42:1, which is comparable to the female-to-male ratio of 2.35:1 in the nationwide Swedish study [33]. However, this sample had a low proportion (47%), of persons with relapsing remitting MS compared to e.g. a large Swedish sample (58.5%, *n* = 16,915) [34]. Fewer persons with relapsing remitting MS answered this survey, possible reasons for this could be that persons with less severe disease did not want to be reminded of the disease, or did not find the questions suitable (e.g. falls self-efficacy scale).

In this cross-sectional study 39-59% of the variation of PA was explained by the regression models. However, this was a cross-sectional study and high correlations are more easily obtained in cross-sectional studies compared to prospective studies. Another disadvantage with cross-sectional studies is that conclusions regarding the causal relationship between different factors cannot be drawn.

The MSIS-29 physical scale and falls self-efficacy scale correlated highly (−0.83 to −0.84), which might have influenced the regression model. However, other controls of multicollinearity were acceptable, and in addition, both factors were considered important to include. The sample size in the subgroup moderate to severe MS was quite small. However, large effects can be detected even in small sample cohorts [22].

One limitation is the use of self-reported measures. We used a subjective self-rated measure of PA, instead of an objective measure, as accelerometer. This was chosen to enable more persons to participate. We also used a self-reported measure of disease impact. However, MSIS-29 is the most widely used patient-reported outcome in MS studies, with the best psychometric properties [35]. Another limitation is that the scale to measure enjoyment of PA is new, and has not yet been psychometrically evaluated.

This study showed that persons with severe disease, those who are unemployed, and those with low self-efficacy for PA are especially prone to inactivity. The importance of self-efficacy for PA, falls self-efficacy and enjoyment is in line with Social Cognitive Theory [12]. According to Bandura [12] self-efficacy is modifiable, and e.g. positive emotions could increase self-efficacy. Strategies for behavioral changes might be applied to increase PA. A review showed that “action planning”, “provide instruction” and “reinforcing effort towards behavior” were behavioral strategies that were associated with higher levels of both self-efficacy and physical activity [36]. Setting a specific detailed plan of when, where and how to perform the physical activity and providing instruction on these same categories, as well as providing positive feedback and reinforce participants efforts in attempting to become more physically active [36], are thus promising ways to promote physical activity in persons with MS. In addition, encouraging enjoyable and pleasurable physical activities, is important to enhance the PA behaviour. Gait, balance and functional training, especially when focusing on challenging balance exercises [27], and fall prevention knowledge, may increase falls self-efficacy, the degree of self-efficacy to avoid a fall.

## Conclusions

This study in Swedish PwMS confirms previous research in other countries regarding the importance of disability level, employment and self-efficacy for PA for the level of PA. This study contributes to previous research in showing that the seldom studied factors falls self-efficacy and enjoyment are also important for PA, and that factors that explain physical activity differs between subgroups with different disease severity. More research preferably in prospective and intervention studies, is needed to confirm the importance of these factors, and to confirm the subgroup differences.

## Abbreviations

ESES: Exercise Self-Efficacy Scale
FES(S): Falls Efficacy Scale Swedish version
FSS: Fatigue Severity Scale
MS: Multiple sclerosis
MSIS-29: Multiple Sclerosis Impact Scale
PA: Physical activity
PADS- R: Physical Activity Disability Survey – Revised
PwMS: Persons with multiple sclerosis
SIPA: Social Influences on Physical Activity
